# Supplementary material for: “The Brain is…”: A Survey of the Brain’s Many Definitions
Source: Neuroinformatics. 2025 Jan 11;23(1):4. doi: 10.1007/s12021-024-09699-x (PMC11724787; doi:10.1007/s12021-024-09699-x)
Supplement: Supplementary file 1 — (DOCX 541 KB) [file 12021_2024_9699_MOESM1_ESM.docx]

**Supplementary Materials**


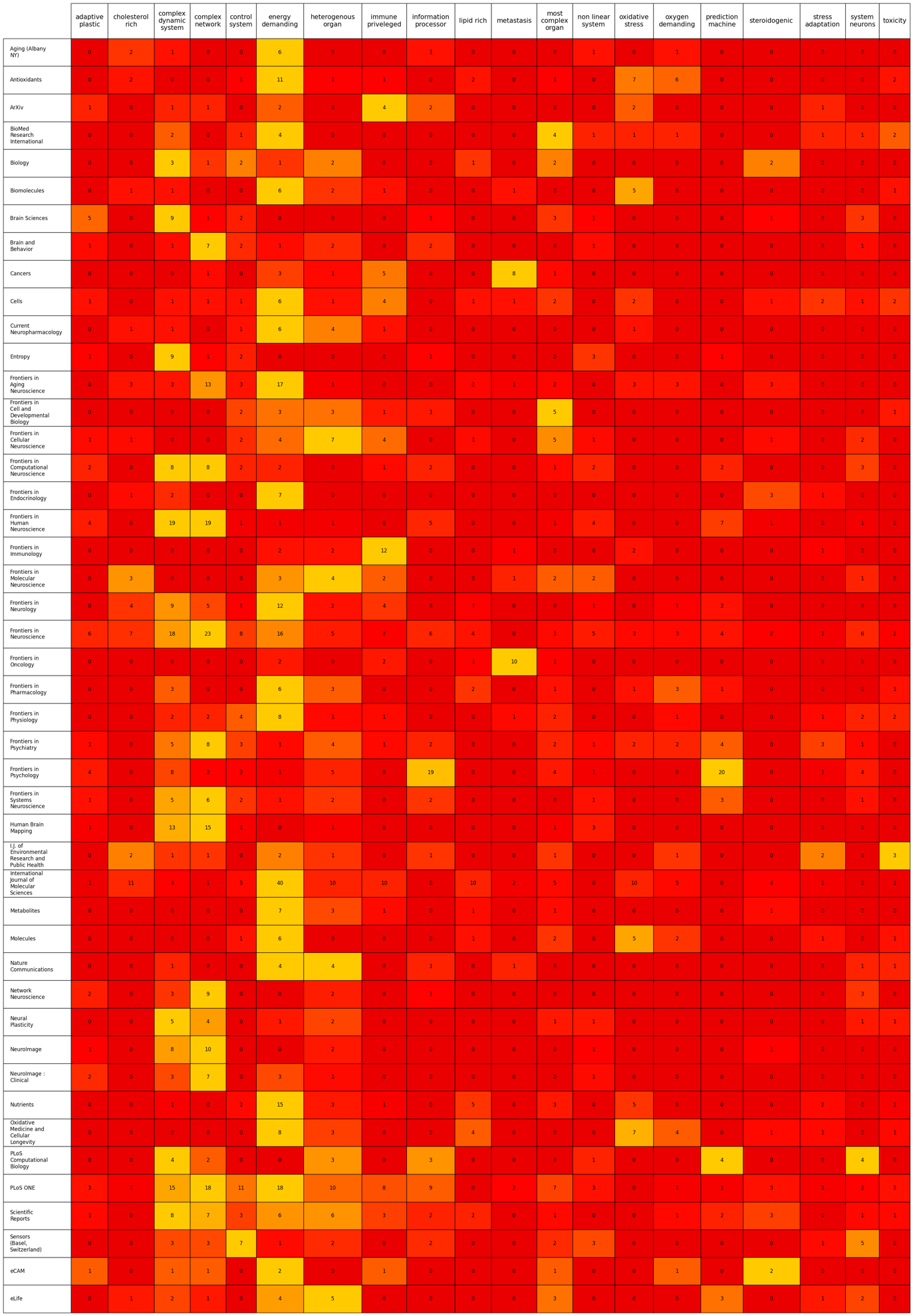


**Supplementary Table 1.** **Counts of Expressions from Each Cluster by Journal Title.** A cross-tabulation table containing the counts of expressions in each cluster (column) by journal title (row). Only journals containing >= 20 expressions were included in the table. Clusters are labeled with the same labels as appears in **Figure 1** (abbreviated for space). Cells of the table are color-coded according to their row-wise normalized (min-max normalization) counts, with lighter colors corresponding to more frequent counts relative to the total counts in each journal (row).

| Journal | Abstract Count |
| --- | --- |
| Journal of Neuroscience | 37967 |
| Journal of Cognitive Neuroscience | 4227 |
| Neuroscience Letters | 35108 |
| Neuropsychologia | 8996 |
| Neuroimage | 22060 |
| Nature Reviews Neuroscience | 1319 |
| Journal of Neurophysiology | 19269 |
| Trends in Cognitive Science | 2316 |
| Neuroscience and Biobehavioral Reviews | 5492 |
| Human Brain Mapping | 5911 |
| Psychophysiology | 4178 |
| Cortex | 4875 |
| Experimental Brain Research | 14804 |
| Nature Reviews Neurology | 888 |
| European Journal of Neuroscience | 13157 |
| Neuron | 11945 |
| The Neuroscientist | 1083 |
| Annual Review of Neuroscience | 696 |
| Molecular Neurobiology | 5933 |
| Brain | 8470 |
| Social Cognitive and Affective Neuroscience | 1980 |
| Nature Neuroscience | 5054 |
| Journal of Neurology | 9330 |
| Behavioral and Brain Sciences | 3465 |
| Progress in Neurobiology | 1921 |
| Biological Psychiatry | 9314 |
| Trends in Neuroscience | 3003 |
| Current Opinion in Neurology | 2644 |
| Cerebral Cortex | 7617 |

**Supplementary Table 2.** **Neuroscience Journal Abstracts Included in Corpus.** The count of abstracts of neuroscience journals included in corpus for analysis.


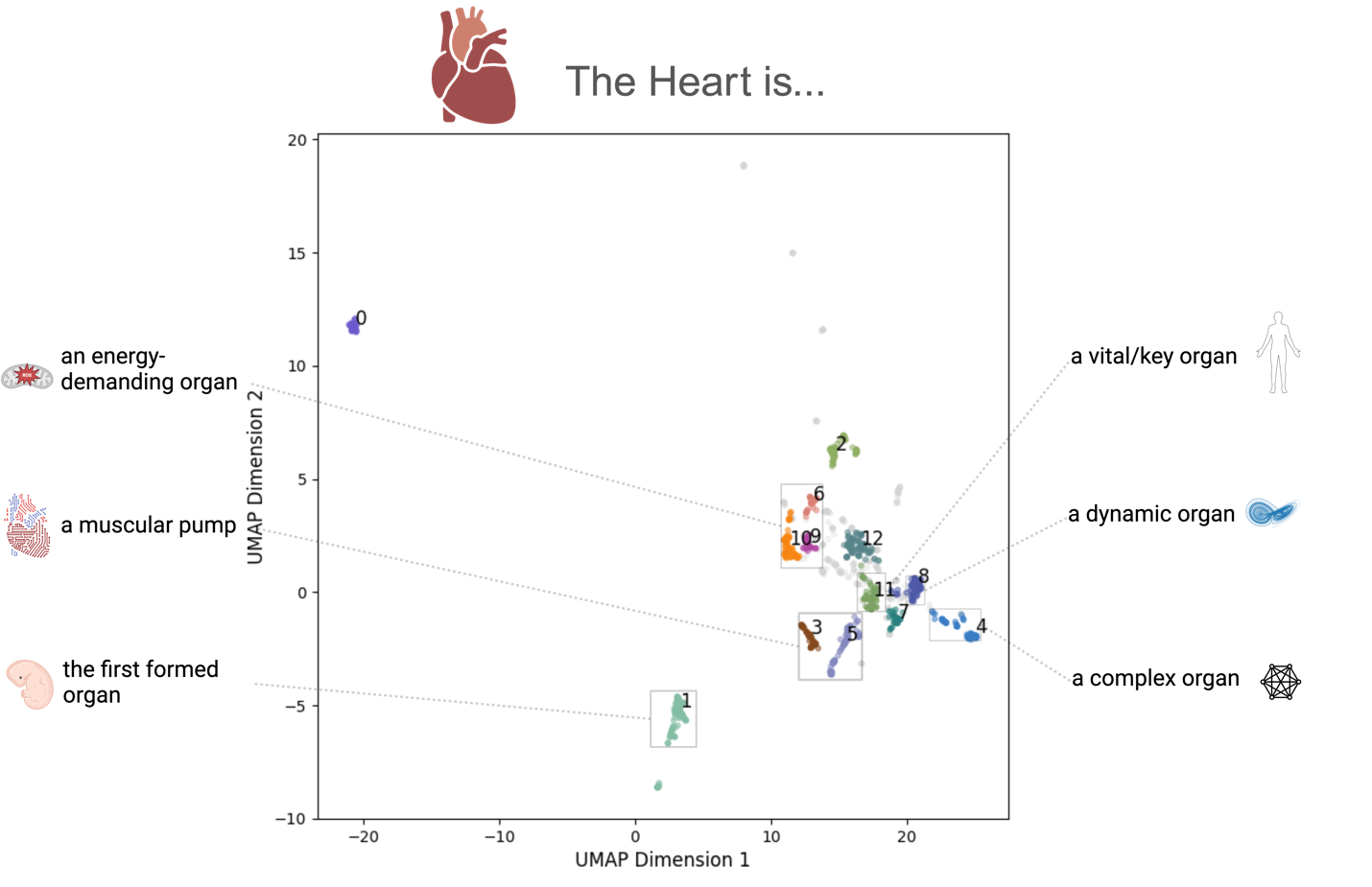


**Supplementary Figure 1**. **‘The heart is…’ Expressions in a Two-Dimensional Embedding Space.** Expressions matching the form ‘The heart is…’ embedded into a two-dimensional space via a dimension-reduction (UMAP) applied to their semantic embeddings. The distance between points in this space reflect the semantic similarity between the expressions – i.e., expressions (points) in this space that are closer together reflect similar meanings. Expressions are color-coded according to their cluster assignment from the HDBSCAN clustering algorithm. Each semantically coherent cluster (N=9) is labeled by a manual interpretation of the expressions in the cluster. Boxes including multiple clusters are mapped to the same label.
